# Supplementary material for: Effect of Nutritional Restriction on the Hair Follicles Development and Skin Transcriptome of Chinese Merino Sheep
Source: Animals (Basel). 2020 Jun 19;10(6):1058. doi: 10.3390/ani10061058 (PMC7341508; doi:10.3390/ani10061058)
Supplement: Supplementary file 1 [file animals-10-01058-s001.zip › Table S1-S4.docx]

**Supplementary Tables:**

**Table S1.** Energy standard and daily feeding intake of experimental ewe in maintenance group.

|  | Ewes  Weight /kg | Pregnancy days of ewes | | | | | | | Lactating days  of ewes | |
| --- | --- | --- | --- | --- | --- | --- | --- | --- | --- | --- |
|  |  | 0 | 30 | 50 | 75 | 100 | 125 | 147 | 7 | 28 |
| Maintenance  Nutrition   requirement  (KJ/per day) | 40 | 7.7 | 7.8 | 7.9 | 8.5 | 9.6 | 11.7 | 14.2 | 15.6 | 16.6 |
|  | 50 | 8.3 | 8.4 | 8.6 | 9.2 | 10.4 | 12.7 | 15.5 | 17.0 | 18.0 |
|  | 60 | 9.0 | 9.1 | 9.5 | 10.1 | 11.3 | 13.8 | 16.8 | 18.4 | 19.4 |
| Daily feed  intake  （kg/per day） | 40 | 0.86 | 0.87 | 0.89 | 0.95 | 1.08 | 1.31 | 1.59 | 1.75 | 1.86 |
|  | 50 | 0.93 | 0.94 | 0.96 | 1.03 | 1.17 | 1.42 | 1.74 | 1.91 | 2.02 |
|  | 60 | 1.01 | 1.02 | 1.07 | 1.13 | 1.27 | 1.55 | 1.88 | 2.06 | 2.17 |

Maintenance nutrition requirement of pregnant ewes refers to the feeding standard of New Zealand Society of Animal Production (NZSAP), In this study, ewes weight between 40 - 60 kg, and ewes were fed a mixed pellet which can provide 8.92 MJ metabolizable energy per kg, Daily feed intake is the amount of maintenance nutrition requirement to the energy of the mixed pellet.

**Table S2.** Changes of blood biochemical indexes in the in the M andsub-M group ewes.

| Time | Groups | TP  (g/L) | ALB  (g/L) | ALT  (IU/L) | AST  (IU/L) | BUN  (mmol/L) | GLU  (mmol/L) | TG  (mmol/L) | HDLC  (mmol/L) | LDLC  (mmol/L) | NEFA  (mmol/L) |
| --- | --- | --- | --- | --- | --- | --- | --- | --- | --- | --- | --- |
| 0d | M | 55.72±11.53 | 22.77±3.73 | 10.48±6.07 | 78.66±12.51 | 5.95±1.80 | 4.34±0.96 | 0.22±0.07 | 0.89±0.13 | 0.47±0.09 | 0.06±0.02 |
|  | Sub-M | 54.44±16.95 | 22.31±4.82 | 8.27±3.23 | 81.14±19.93 | 5.17±1.92 | 3.13±0.75 | 0.18±0.07 | 0.80±0.15 | 0.50±0.19 | 0.09±0.04 |
| 7d | M | 61.24±8.26 | 24.7±2.23 | 9.36±3.29 | 72.69±36.42 | 5.53±1.97 | 3.37±0.50 | 0.2±0.07 | 0.95±0.12 | 0.48±0.11 | 0.12±0.08 |
|  | Sub-M | 63.51±4.80 | 24.89±2.71 | 8.65±2.95 | 70.24±14.80 | 6.06±1.02 | 3.53±0.86 | 0.2±0.07 | 0.89±0.21 | 0.57±0.13 | 0.09±0.04 |
| 14d | M | 67.1±8.50 | 26.35±1.50 | 12.42±5.93 | 97.47±39.52 | 5.07±2.18 | 4.17±0.30 | 0.26±0.10 | 1.01±0.12^a^ | 0.69±0.23 | 0.07±0.03 |
|  | Sub-M | 60.05±7.70 | 23.79±2.51 | 7.67±3.85 | 74.08±17.59 | 4.83±1.12 | 4.04±0.67 | 0.22±0.08 | 0.81±0.13^b^ | 0.62±0.11 | 0.05±0.02 |
| 21d | M | 64.8±5.48 | 26.16±0.70 | 9.68±2.24 | 87.19±45.79 | 4.05±1.26 | 4.58±0.56 ^b^ | 0.27±0.05 | 1.05±0.10^a^ | 0.53±0.12 | 0.10±0.03 |
|  | Sub-M | 62.59±5.13 | 24.81±2.22 | 9.08±3.74 | 117.59±28.09 | 5.35±0.96 | 5.31±0.57^a^ | 0.31±0.09 | 0.8±0.16^b^ | 0.59±0.16 | 0.09±0.04 |
| 28d | M | 65.7±85.35 | 26.56±1.21 | 8.54±2.23 | 95.46±49.24 | 8.64±1.13 | 4.28±0.33 | 0.23±0.02 | 1.15±0.14^a^ | 0.58±0.13 | 0.09±0.02^b^ |
|  | Sub-M | 63.08±6.68 | 24.8±2.111 | 11.27±6.88 | 148.93±67.59 | 8.45±1.31 | 3.77±0.68 | 0.19±0.07 | 0.87±0.17^b^ | 0.65±0.10 | 0.15±0.02^a^ |

Ewes blood biochemical were assayed every 7 days during 105-135 days of gestation.TP: Total protein, ALB: Albumin, ALT: Alanine aminotransferase, AST: Aspartate aminotransferase, BUN: Urea nitrogen, GLU: Blood glucose, TG: Triglyceride, HDLC: High-density lipoprotein, LDLC: Low-density lipoprotein, FFA: Free fatty acids.
Data are means± standard deviation(SD). ^a,b^ Within column, different superscript letter indicates differences (p < 0.05) between M and Sub-M group at the same time point.

**Table S3.** Primer information of qRT-PCR

| Gene | sequence(5' to 3') |
| --- | --- |
| PITX2-F | GCGGACACTAAAGAAAGAAAGG |
| PITX2-R | TCCAGTCAAACATTTGTGTGTC |
| BMP5-F | AGAGACAAAGATTTTTCGCACC |
| BMP5-R | GCTCCTATCCTTGTATATCCGG |
| BMP3-F | ATTTCATGTTCTGACAAGCGTC |
| BMP3-R | GGTAACTCTTGCATACCCTCTT |
| IGF2BP2-F | GAACTGCAGAACTTAACCAGTG |
| IGF2BP2-R | GCCCGATAATTCTGACAATCAC |
| BMP2-F | ATCACCTGAACTCCACGAA |
| BMP2-R | TACCACCTTCTCATTCTCATC |
| KRT16-F | CTACCTGAAGAAGAACCACGAG |
| KRT16-R | TGTCTGCTATCTGCTCATACTG |
| NOG-F | CCAGCACTATCCCCACATCC |
| NOG-R | CCATGAATCCCGGGTCGTAG |
| PITX1-F | CAAGAGCTTCACCTTCTTCAAC |
| PITX1-R | GTGAGGTTGTTGATGTTGTTGA |
| GAPDH-F | GCGACACTCACTCTTCTACCTTC |
| GAPDH-R | TCTCTTCCTCTCGTGCTCCTG |

**Table S4.** Comparison of fetal body size of the M and Sub-M fetuses at different stages of gestation.

| Category | 85 days of gestation | | 105 days of gestation | | 135 days of gestation | |
| --- | --- | --- | --- | --- | --- | --- |
|  | M1(n=3) | Sub-M1(n=3) | M2(n=3) | Sub-M2(n=3) | M3(n=3) | Sub-M3(n=3) |
| Body length(mm) | 24.44±0.60 | 25.36±1.20 | 31.33±0.83 | 31.25±0.25 | 50.33±1.33 | 45.83±3.19 |
| Body height(mm) | 16.67±0.30 | 16.00±0.80 | 23.67±0.44 | 22.50±0.50 | 40.33±0.88 | 34.83±0.17 |
| Chest circumference(mm) | 16.67±0.54 | 18.50±2.12 | 22.00±0.53 | 21.50±0.50 | 34.33±0.57 | 35.17±0.44 |
| Abdomen   Circumference(mm) | 13.70±0.15 | 17.80±0.75 | 22.10±0.78 | 23.50±0.50 | 29.67±2.40 | 31.60±1.33 |
| Weight of fetal   Sheep(g) | 458.00±18.61 | 486.00±60.00 | 1095.00±57.44 | 888.00±50.00 | 4003.00±144.36 | 3536.00±176.67 |
| Heart weight(g) | 4.44±0.27 | 4.37±0.38 | 10.50±0.60 | 9.90±0.60 | 36.3±0.30 | 31.20±2.90 |
| Liver weight(g) | 24.40±2.70 | 25.40±0.60 | 59.80±2.30 | 47.90±4.60 | 104±9.70 | 89.08±4.50 |
| Spleen weight(g) | 0.65±0.10 | 0.85±0.21 | 2.20±0.40 | 1.50±0.00 | 7.00±0.30 | 5.30±0.60 |
| Lung weight(g) | 25.40±0.77 | 22.50±2.15 | 55.96±2.01 | 48.25±5.10 | 172.30±17.50 | 144.60±9.90 |
| Kidney weight(g) | 6.40±0.03 | 5.40±0.60 | 11.90±1.90 | 9.70±0.60 | 44.70±7.80^a^ | 29.07±4.10^b^ |

Data are means±SD.^ab^ Within a row, different superscript letter indicates differences (p < 0.05) at the same gestation stages.
