# Supplementary material for: Effect of Nutritional Restriction on the Hair Follicles Development and Skin Transcriptome of Chinese Merino Sheep
Source: Animals (Basel). 2020 Jun 19;10(6):1058. doi: 10.3390/ani10061058 (PMC7341508; doi:10.3390/ani10061058)
Supplement: Supplementary file 1 [file animals-10-01058-s001.zip › Figure S1 Validation of DEGs by qRT-PCR.docx]

| 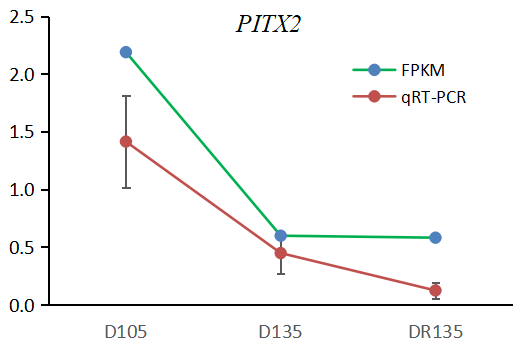 | 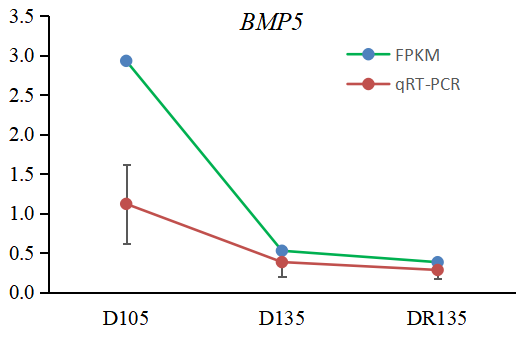 |
| --- | --- |
| 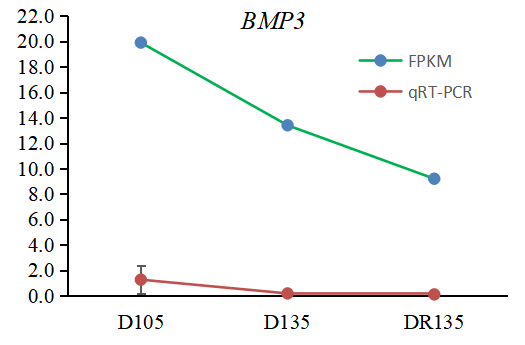 | 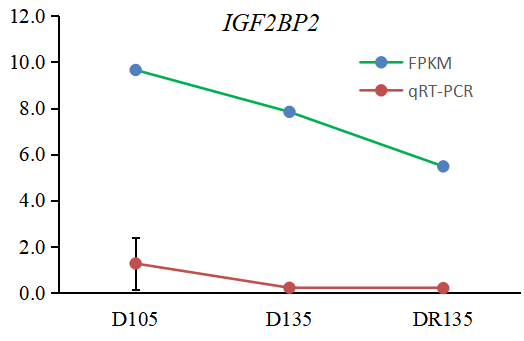 |
| 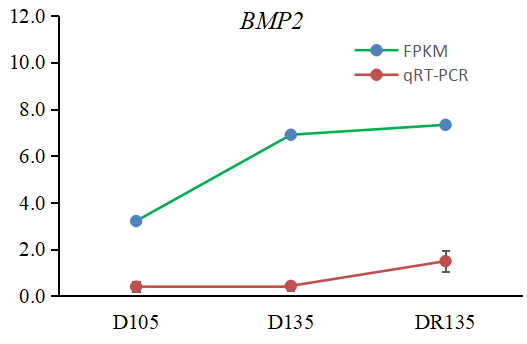 | 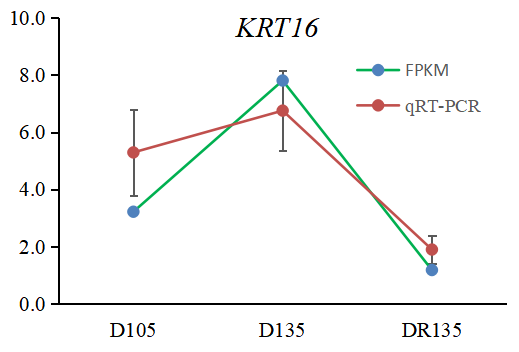 |
| 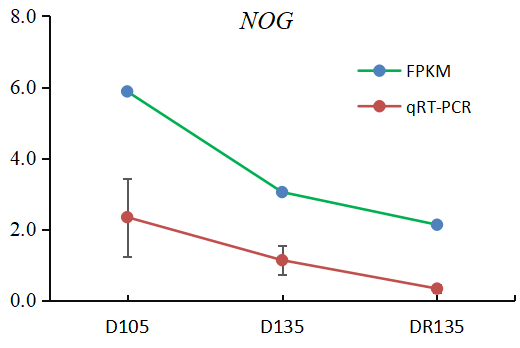 | 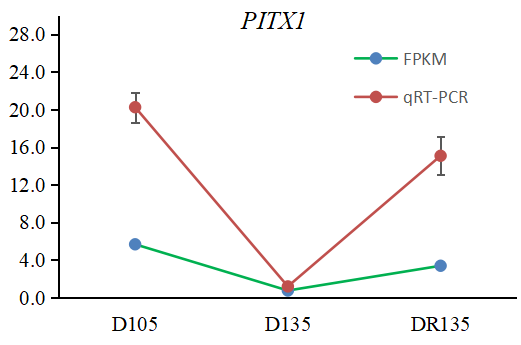 |

**Figure S1**: qRT-PCR validation of the 8 DEGs.The x-axis represents the three developmental stages, The y-axis represents the gene expression level. Expression levels were normalized to the expression level of GAPDH. Error bars SE from three independent experiments.
